# Gamification, a Successful Method to Foster Leptospirosis Knowledge among University Students: A Pilot Study

**DOI:** 10.3390/ijerph16122108

**Published:** 2019-06-14

**Authors:** Nurul Natasya Azhari, Rosliza Abdul Manaf, Shing Wei Ng, Siti Farhana Bajunid Shakeeb Arsalaan Bajunid, Abdul Rahman Mohd Gobil, Wan Zuhainis Saad, Syafinaz Amin Nordin

**Affiliations:** 1 Department of Medical Microbiology and Parasitology, Faculty of Medicine and Health Sciences, Universiti Putra Malaysia, Serdang 43400 UPM, Selangor, Malaysia; natasyazhari99@gmail.com; 2 Department of Community Health, Faculty of Medicine and Health Sciences, Universiti Putra Malaysia, Serdang 43400 UPM, Selangor, Malaysia; rosliza_abmanaf@upm.edu.my; 3 Institute of Bioscience, Universiti Putra Malaysia, Serdang 43400 UPM, Selangor, Malaysia; shingwei89@gmail.com; 4 Department of Veterinary Clinical Studies, Faculty of Veterinary Medicine, Universiti Putra Malaysia, Serdang 43400 UPM, Selangor, Malaysia; 5 Faculty of Biotechnology and Biomolecular Sciences, Universiti Putra Malaysia, Serdang 43400 UPM, Selangor, Malaysia; farhanabajunid@gmail.com (S.F.B.S.A.B.); zuhainis@upm.edu.my (W.Z.S.); 6 Department of Computer Science, Faculty of Computer and Mathematical Sciences, Universiti Teknologi MARA, UiTM Cawangan Negeri Sembilan, Kampus Seremban, Seremban 70300, Negeri Sembilan, Malaysia; argobil@uitm.edu.my; 7 Medical Education Research and Innovation Unit, Faculty of Medicine and Health Sciences, Universiti Putra Malaysia, Serdang 43400 UPM, Selangor, Malaysia

**Keywords:** youth, leptospirosis, awareness, knowledge, gamification

## Abstract

Leptospirosis is a zoonotic disease that has been reported in Malaysia and has been associated with a recent trend of recreational activities among the youth. Thus, efforts such as educational interventions among high-risk populations, especially the youth, are key to increasing public awareness regarding leptospirosis. This paper presents the findings of a pilot study wherein an educational intervention using a gamification intervention method was used to determine changes in leptospirosis knowledge among youth. On this note, students from a public university in Seremban district, Malaysia, were recruited and were asked to complete questionnaires before and after gamification activities. Baseline and immediate post-intervention data on leptospirosis knowledge were obtained. The total knowledge score was calculated, and differences in the mean pre- and post-intervention knowledge score were determined. Of the total 185 questionnaires that were completed at baseline and immediately post-intervention, only 168 that belonged to respondents who had heard of leptospirosis were analysed in this paper. A significant increase in leptospirosis knowledge was observed for the students following health education by gamification (*p* < 0.01). The results demonstrate the effectiveness of an educational intervention using gamification in improving leptospirosis knowledge among youth and suggest that gamification could become an efficient tool to prevent the disease within university-age demographics.

## 1. Introduction

Leptospirosis is a zoonotic disease that was designated as a notifiable disease in 2010 in Malaysia [1]. It has raised concerns owing to the increasing number of cases reported every year, with a mortality rate > 10%, particularly in Southeast Asia and America [2,3]. The National Notifiable Disease Surveillance System estimated 1.03 million cases of infections and 58,900 human deaths every year worldwide, with an annual average of 0.55 notified cases/100,000 individuals; moreover, tropical countries are hotspots, accounting for more than half of the global estimated cases [4]. Leptospirosis is caused by the pathogenic species of *Leptospira,* primarily *Leptospira interrogans*. Till date, 38 species of *Leptospira* have been identified: 13 pathogenic, 12 intermediates, and 13 non-pathogenic (saprophytes) [5,6]. *L. interrogans* persistently colonizes the proximal renal tubules of carrier animals, and, once infected, the carrier animals remain symptom-free and shed infectious organisms in urine for their entire lifetime [7]. Although all mammals can carry this pathogen in their kidneys, rodents are responsible for most cases of leptospirosis in humans [8]. Despite being incidental hosts, humans exhibit acute to severe symptoms following infection [9,10]. Infection in humans occurs when the bacterium is transmitted into the bloodstream following direct contact with an infected animal or an indirect contact with water and soil contaminated with the urine of an infected animal [11]. The bacterium invades through cuts and abrasions or mucous membranes such as the conjunctival, oral, or genital surfaces [7].

The risk of developing leptospirosis, which was associated with occupational activities such as livestock farming, veterinary medicine, sewer maintenance, butchering, and military activities a decade ago [12], has re-emerged, with prevalence in tropical and sub-tropical countries. Currently, recreational activities and ecotourism are the leading causes of leptospirosis in the young population [13,14]. Furthermore, the risk of developing leptospirosis in a particular area is suggested to be associated with the environmental and socio-economic factors of that area [15,16]. Developing tropical countries are considered to be the ideal habitats for the survival of *Leptospira* species; moreover, the occurrence of the disease exacerbates during heavy rainfall and floods, with the most notable outbreaks being in Nicaragua (1995), Peru and Ecuador (1998), Orissa (1999), Malaysia (2000), Jakarta (2002), Mumbai (2000 and 2005), and The Philippines (2009) [17]. High exposure has been recently observed at recreational areas with access to waterfalls and wetlands and those close to forest reserves, which are commonly used by people for vacationing or camping [18,19,20]. Exposure is more intense in such areas owing to the accumulation of garbage piles and litter after visits by people, as well as in unsanitary environments, especially after flooding and heavy rainfall. This environment lures rat populations around the deposited garbage in search of food and thus increases the chance of human contact with infected rats [21].

In Malaysia, some incidents of leptospirosis that occurred throughout the year and were associated with recreational and water-based activities include an outbreak in participants of the Eco-Challenge in Borneo [14], an outbreak associated with swimming in Beaufort [22], an outbreak of melioidosis co-infection [23], and the death of a national service trainee because of suspected leptospirosis [24]. These were followed by a few, recent, sporadic cases reported in the national daily news; press releases by the officials of the Ministry of Health addressed the deaths of two students swimming in contaminated rivers in Jeram and a waterfall in Gunung Pulai in the year 2017 and another case involving a student infected with leptospirosis after a visit to the recreational park in Jelebu [25]. Thus, highlighting recreational exposure as a determining factor in the transmission of leptospirosis is crucial. The Ministry of Health also reported that outbreaks associated with recreational exposure accounted for 32% and 22% in the last three years [26]. Areas in Peninsular Malaysia such as Selangor and Kelantan are among the worst infected areas, with 365 cases, while Sarawak and Terengganu had 362 and 323 cases, respectively [27].

**Figure 1:**
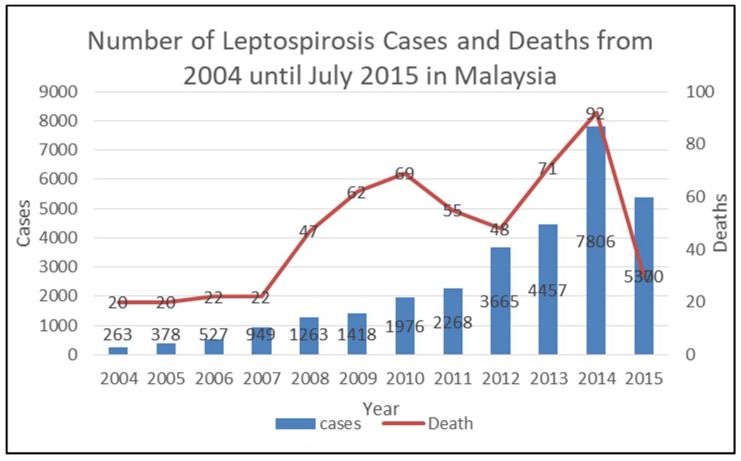
Summary of the number of leptospirosis cases and associated deaths in Malaysia from 2004 to July 2015 [26].

Although the disease became reportable in 2010 in Malaysia [28], the number of cases and fatalities continued to increase dramatically, with the highest number of cases reported in 2015 (Figure 1). Statistics show that 4457 cases were reported in 2013, and the proportion of patients aged ≤ 19 years was among the highest with 23.3%, followed by those of patients aged 25–29 years, with 13%, and 20–24 years with 12.1% [29]. Analyzing the data according to professions revealed that students (16.9%) have a high risk of contracting leptospirosis, followed by agriculture-based workers (14.7%) [29]. These findings are in concordance with the trend in China, where 18–22% of patients with leptospirosis were reported to be students [30]. This high prevalence could be because of the recent trend of recreational activities that are frequently performed by students aged 19–25 years. Thus, intervention and control measures need to be implemented to prevent further incidents in this age group.

Gamification uses game thinking and mechanics in non-game contexts to engage users in solving problems [31]. This method is now becoming a blooming trend in learning to encourage specific behaviours and increase motivation and engagement [32]. Previous studies demonstrated the usefulness of disease prevention, health promotion, and increased learning through gamification [33,34]. A few intervention studies were conducted in Malaysia, specifically targeting populations at high risk of contracting leptospirosis such as town service and wet-market workers and also rural communities [35,36,37]. However, to the best of our knowledge, no intervention programmes have been conducted for students or younger populations. Thus, this pilot study was conducted to assess the effectiveness of an intervention programme that uses gamification as a tool to instil leptospirosis knowledge among students. The game-based method was selected because it is a powerful method for achieving student engagement in learning [38].

## 2. Materials and Methods

### 2.1. Study Design, Sampling Population, and Sample Size

This was a pilot study of an experimental before and after study conducted during a four-hour session in July 2017. A simple random sampling technique was employed to collect a sample of 185 students aged <25 years, enrolled in a public university of Seremban district, Malaysia. This sample size is sufficient for a pilot study as suggested by Whitehead et al. for a main trial designed with 90% power and two-sided 5% significance [39]. The district of Seremban was chosen as the sampling area as there were a few cases of leptospirosis reported involving students in the year of the study period.

### 2.2. Inclusion Criteria

Students aged <25 years enrolled in the public university of Seremban, with no background in health sciences were included in the study. They were local students who agreed to participate in the study.

### 2.3. Study Instrument

#### Questionnaire

The questionnaire and the scoring system were adapted and modified on the basis of items listed from a previous study on leptospirosis in Malaysia [40]. For the knowledge scale, one item was removed from the previous study [40], which is “The disease can be prevented by taking a bath after working”, and four items were included (risk reduction, rodent control, food storage, and footwear) (supplementary Table S1). Following the pre-test on 30 respondents, the internal consistency of the knowledge scale was found to be adequate, with a Cronbach-alpha value of 0.80. This is in line with the recommendation of adequate Cronbach alpha value (>0.70) [41]. The self-administered questionnaire was developed in Malay language and was divided into four sections which included demographic data, knowledge, attitude, and practice. The knowledge section focused on symptoms, transmission, treatment, and the prevention of leptospirosis. This paper only focuses on the changes in the knowledge component following the gamification intervention. Hence, attitudes and current practices regarding the disease were not included in the analysis. There were 25 knowledge questions in which the respondents were given 3 options to answer, which were “Correct”, “Wrong”, and “Do not know”. It started with a question asking whether the respondents had ever heard of leptospirosis, and they were asked to specify the source of their information. Those who had ever heard of the disease proceeded to answer the rest of the knowledge questions. Each correct answer was scored as 1, while a 0 score was given to a wrong answer and to a “do not know answer”, respectively. The total knowledge score was calculated, and the differences in the mean knowledge scores pre- and post-intervention were determined.

#### Gamification Module

The module comprised 10 stations: station 1 (Puzzle), station 2 (Find *Leptospira* in the flour), station 3 (Fishing using chopsticks), station 4 (Dart), station 5 (Doodle), station 6 (Wink-wink leptospirosis infection), station 7 (Charades), station 8 (Instagram), station 9 (Parody), and station 10 (Role-playing game) (Table S2). The students were asked to complete all stations before completing the post-evaluation questionnaire.

This educational intervention module was designed to test the creativity and speed of the participants in solving problems as well as to teach them regarding leptospirosis. Students were exposed to five aspects in this module: symptoms, transmission, treatment, prevention, and the characteristics of *Leptospira* and its infection. Ten stations were used, each with different activities and tasks, during the intervention programme. These stations presented different educational aspects regarding leptospirosis for the students to learn and play. For example, station 1 (Puzzle) focused on the characteristics of *Leptospira*, station 2 (Find *Leptospira* in the flour) taught the participants regarding the shape of *Leptospira*, station 3 (Fishing chopstick) focused on the symptoms, station 4 (Dart) focused on transmission, station 5 (Doodle) focused on prevention, station 6 (Wink-wink leptospirosis infection) showed how leptospirosis is transmitted, station 7 (Charades) focused on treatment, station 8 (Insta) focused on symptoms, station 9 (Parody) recapped the information gathered regarding the disease as a whole, and station 10 (RPG game) addressed the symptoms, treatment, and prevention.

Participants required at least 1 min to complete each station depending on the task given. Some stations required up to 11 min. Students were divided into 10 groups with an average of 19 students per group. The groups were placed at each station according to their group number and had to complete all 10 stations. For example, group 1 started at station 1, and group 2 started at station 2. After a group completed the task at their assigned station, they went to any station they wanted until they completed all 10. The activity started by giving them the amount of time they needed to complete the task. Once finished, the scores were collected. After completing all 10 stations, the total scores were calculated, and the group with the highest scores was announced as the winner.

### 2.4. Data Collection

Eligible respondents were recruited and were asked to complete a questionnaire to provide data at the baseline. Next, the students were divided into groups of 10. Each group performed the gamification activities. After completion of the intervention, the respondents were asked to repeat the questionnaire on leptospirosis knowledge.

### 2.5. Data Analysis

Data were organised and analysed using the SPSS Version 23 statistical software (SPSS Inc., Chicago, IL, USA). Descriptive statistics were used to determine socio-demographic factors. A normality test using box-plots and the Kolmogorov–Smirnov test showed that the knowledge scores were not normally distributed [42]. Therefore, the Wilcoxon signed ranks test was conducted to identify the difference in knowledge scores pre- and post-intervention. The significance level was set to *p* < 0.05.

### 2.6. Study Ethics

Written consent was obtained from all respondents for the study protocol (reference number JKEUPM(FPSK-P126)2017) that was approved by the Ethics Committee for Research Involving Human Subjects (JKEUPM), Universiti Putra Malaysia.

## 3. Results

### 3.1. Demographic Characteristics

From the study, a total of 185 questionnaires were completed at baseline and immediately post-intervention. However, only 168 questionnaires that belonged to respondents who had heard of leptospirosis were analysed to suit the aim of this paper to determine the usefulness of gamification in increasing participants’ knowledge. Seventeen questionnaires that belonged to those who had never heard of leptospirosis were excluded from this analysis to reduce information bias in term of intelligent guess. The demographic background of the 17 respondents who were excluded from the study consisted mostly of female (13/17), within the age range of 18–20. Table 1 shows the demographic characteristics of the 168 participants who were included in data analysis. The age of the participants ranged from 18 to 23 years, with a mean (SD) of 18.7 (0.9). The participants were primarily females (155, 92.3%), and 13 (7.7%) participants were men. The majority of the participants were Malay (164, 97.6%). More than half (93, 55.4%) lived in terrace houses; residents of flats, apartments/condominiums, bungalows, and other house types accounted for 8.9%, 3.0%, 20.2%, and 12.5% participants, respectively.

### 3.2. Awareness and Source of Information on Leptospirosis

From the findings, 90.8% (168/185) of the respondents had heard about leptospirosis before the study. The remaining 9.2% (17/185) respondents had never heard about leptospirosis. Most of the respondents stated that television/radio and online news were their main sources of information.

### 3.3. Leptospirosis Knowledge

From the study findings, a statistically significant increase (*p* < 0.01) was noted in mean knowledge scores immediately post-intervention compared with the scores at the baseline. Table 2 shows the list of questions asked to the 168 respondents who had heard about leptospirosis before the intervention along with the percentage scores of correct answers in the questionnaire pre- and post-intervention. More than half of the participants obtained good scores (>63%) in knowledge related to leptospirosis before the intervention, with a mean percentage of 65.2% for the total knowledge score. The immediate effects of the education intervention were observed as the percentage of correct answers post-intervention increased to a mean of 85.2%. Most of the students correctly identified leptospirosis as a zoonotic disease (82.1%) and knew that leptospirosis is caused by microorganisms (71.4%). Eating contaminated food was the most well-known route of transmission, with a response of 68.5%. However, only 32.7% of the participants knew that the bacteria can enter through wounds. Surprisingly, shaking hands with an infected person was considered one of the routes of transmission (42.3%). Regarding the signs and symptoms of leptospirosis, 62.5% of the respondents knew muscle ache (myalgia) was a symptom of the disease, and 92.9% knew that a lack of treatment may lead to death. However, only 26.2% of the participants knew that infected persons may experience yellow fever (jaundice). As for the complications, only 40.5% and 33.3% of the participants knew that leptospirosis can lead to kidney failure and liver damage.

Although a majority of the respondents (72.6%) knew that municipal council workers are included in the high-risk group, most were not aware that drinking while working may contribute to the risk of infection, with only 17.3% responding correctly. Meanwhile, with regard to the treatment and preventive measures for leptospirosis, most respondents (81.3%) knew that leptospirosis can be treated (71.4%) and detected through blood tests (89.9%). Furthermore, they knew that the disease can be prevented by maintaining cleanliness in surrounding areas (97.6%), followed by eliminating rodents and breeding areas (93.5%), storing food in a proper manner (89.3%), reducing high-risk activities associated with leptospirosis (82.7%), avoiding walking in water puddles or areas of frequent flooding (81.0%), avoiding walking barefoot (63.1%), and wearing gloves while working (60.1%).

## 4. Discussion

Leptospirosis has re-emerged as a tropical disease with global burden, and its transmission is associated with a variety of ecological factors ranging from unsanitary environment and improper waste management to natural disasters (flooding), leading to substantial outbreaks [25,43,44]. Thus, deliberate attempts to improve community awareness and understand the risk factors and prevention measures, particularly in endemic areas, is essential for public health education regarding leptospirosis [36,45,46].

### 4.1. Awareness and Knowledge of Leptospirosis Regarding Infection Sources and Modes, Risk Factors, and Disease Treatment and Prevention

This pilot study was designed to assess the effects of the gamification module on changes in knowledge regarding leptospirosis. Thus, university students were selected randomly to tackle the knowledge gaps regarding leptospirosis, primarily on infection sources and modes, risk factors, and disease treatment and prevention. The attitudes and current practices regarding the zoonotic importance of the disease were not studied. Interestingly, a low percentage of respondents had never heard of leptospirosis (9.2%). A large percentage of people are aware of the disease probably because of the extensive media coverage on recent outbreaks of leptospirosis related to recreational activities and flooding in Malaysia [44,47,48]. Similar studies have also reported a very high percentage of respondents who had heard of leptospirosis [49,50]. According to the present study, 90.8% of interviewed students who were familiar with leptospirosis obtained the information primarily from mass media such as online news, radio, and social media. Furthermore, the demographic characteristics revealed that most of the students lived in terrace houses, which are considered urban houses in well-managed towns with proper sanitary conditions. This may also have contributed to the high percentage of knowledge regarding the disease, as a high risk of infection can be observed among people living in urban slums and unsanitary environments [51]. The location of the university (considered as an urban area) can also be attributed to the higher percentage of students who had leptospirosis knowledge, as they had easy access to different media and sources from the internet. This shows that mass media are a powerful tool to disseminate health news to a community, and increasing the promotion of health campaigns through media coverage is an effective awareness strategy [46,52].

In addition to the demographic characteristics, a gender imbalance was observed, as a majority of female respondents (92.3%) were involved in the study. This is not surprising because female students perform better than male students in Malaysia’s public education system; thus, the number of female students enrolled in universities is higher than that of male students [53]. The high number of females involved in the awareness programme may also be due to the fact that females are more health-conscious than males. This has been demonstrated in previous studies where men tended to be unaware of the sources of health-related information and have inadequate competence to be active seekers of health issues owing to pure ignorance or reluctance, low motivation, and a tendency to seek out what they know to be available [54,55].

On the basis of the answers provided, a majority of respondent knew about leptospirosis infection sources and modes. However, a low percentage of the respondents, both pre- and post-intervention, were aware that untreated leptospirosis may lead to kidney failure and liver damage. The respondents were also unaware that infected persons may experience yellow fever. This finding indicates the importance of educating individuals regarding the severity of untreated leptospirosis in humans in future interventions.

Half of the students knew that municipal council workers are among those in the high-risk group; however, the majority was not aware that drinking while working can also result in infection. Knowledge regarding the risk factors of infection is of utmost importance in preventing leptospirosis [40]. Most of the respondents obtained good scores for the knowledge on disease treatment and prevention. This is not surprising, as most people believe that maintaining personal hygiene can prevent all types of infections [52,56,57,58].

### 4.2. Gamification Module

The findings of the current study showed a significant increase in the knowledge of the students regarding leptospirosis following health education by gamification (*p* < 0.01). The success of using gamification as a tool to promote health education by involving the participants in various physical activities was also demonstrated in earlier studies [45,58]. Gamification is a promising activity that helps people maintain and recall knowledge in interactive ways due to increased attention, commitment, and motivation. Moreover, the approach of using a gamification module to disseminate health information can also be adapted to high-risk communities apart from the university population, albeit with some modifications that are better suited to the targeted populations.

Numerous physical activities were employed during the intervention for the effective delivery of health education content. Role play, games (puzzles and darts), goal-oriented activities, and social media engagement were used as a medium to convey the message. This enabled everyone to participate and give their opinions on the subject matter and also better understand the practical preventive measures compared with listening to lectures or video presentations. Our study is also consistent with earlier intervention studies on leptospirosis, which concluded that educational intervention programmes have the strongest effect on improving leptospirosis knowledge, particularly in high-risk populations [36,46]. The usefulness of digital games in the study could also be compared with other studies that highlighted opportunities for disease prevention, health promotion, and increased learning [33,34]. Therefore, the present study confirms that an educational intervention using the gamification method has a positive impact on enhancing leptospirosis knowledge among university students and similar-age groups. Moreover, social media was the preferred source of information and was the source that allowed the students to obtain most of their knowledge regarding the disease.

### 4.3. Limitations

This study has several limitations that need to be highlighted. The gamification module that was established could not be broadly applied to all populations. A sub-analysis of the module should be conducted to determine whether its contents can be applied to other populations, particularly high-risk populations. To modify the module, another intervention programme with the same contents should be conducted to address different learning styles in participants of difference demographic backgrounds. Other than that, the post-test for the present study was immediately conducted after the gamification activity, and no post intervention follow-up was conducted on the same group of respondents after the study. Thus, a post-intervention follow-up should be conducted to see whether the knowledge is retained.

## 5. Conclusions

In conclusion, a leptospirosis health intervention using a gamification method was demonstrated to effectively improve leptospirosis knowledge among university students. Mutual involvement between the volunteers and health professionals should be encouraged to impart knowledge as well as to strategize and develop good action plans or preventive modules for leptospirosis. Therefore, gamification can be a useful strategy for health education of the youth and other high-risk groups to improve their knowledge regarding leptospirosis and their health consciousness in their immediate environments.

## Figures and Tables

**Table 1:** Demographic characteristics of the participants (n = 168).

Variable	n (%)
Age in years, mean (SD)	18.7 (0.9)
(18–23 years old)	
Sex	
Male	13 (7.7)
Female	155 (92.3)
Ethnicity	
Malay	164 (97.6)
Non-Malay	4 (2.4)
House type	
Flat	15 (8.9)
Apartment/Condominium	5 (3.0)
Terrace house	93 (55.4)
Bungalow	34 (20.2)
Others	21 (12.5)

**Table 2:** List of questions asked to the respondents who had heard about leptospirosis during the gamification intervention, with the percentage scores for correct answers pre- and post-intervention (n = 168).

Questions	n = 168	
	% of Correct Answers before Education by Gamification	% of Correct Answers after Education by Gamification
Infection source and mode		
Leptospirosis is a disease caused by a microorganism	71.4	89.1
It is a zoonotic disease	82.1	97.8
It can be transmitted through wounds	32.7	94.0
It can be transmitted through contaminated food	68.5	95.1
It can be transmitted by mosquito bites	70.2	90.2
Individuals may be infected if they shake hands with infected people	57.7	77.7
Clinical signs, symptoms and complications		
Infected individuals will experience muscle ache (myalgia)	62.5	98.9
Infected individuals will experience yellow fever (jaundice)	26.2	96.7
Infected individuals are free from any disease symptoms	74.4	87.0
The disease may cause death	92.9	97.8
The disease may cause lung cancer	45.8	80.4
The disease may cause kidney failure	40.5	38.0
The disease may cause liver damage	33.3	33.7
The disease may cause diabetes	59.5	87.5
Risk		
Drinking while working increases the risk of leptospirosis	17.3	65.8
Municipal council workers are not included in high-risk group	72.6	75.5
Treatment and prevention		
This disease can be treated	71.4	91.3
This disease can be detected through blood tests	89.9	90.2
Maintaining the cleanliness of surrounding areas may prevent the disease	97.6	100
Avoiding walking in water puddles or areas of frequent flooding can prevent this disease	81.0	98.4
Wearing gloves while working may prevent the disease	60.1	92.4
Reducing high-risk activities associated with leptospirosis prevents the disease	82.7	97.8
Eliminating rodents and breeding areas is a preventive measure for the disease	93.5	98.4
Storing food in a proper manner can prevent the disease	89.3	96.2
Avoiding walking barefoot can prevent the disease	63.1	96.7

## Supplementary Materials

The following are available online at https://www.mdpi.com/1660-4601/16/12/2108/s1, Table S1: Questionnaire, Table S2: Gamification module description.
