# Supplementary material for: Gamification, a Successful Method to Foster Leptospirosis Knowledge among University Students: A Pilot Study
Source: Int J Environ Res Public Health. 2019 Jun 14;16(12):2108. doi: 10.3390/ijerph16122108 (PMC6616438; doi:10.3390/ijerph16122108)
Supplement: Supplementary file 1 [file ijerph-16-02108-s001.pdf]

S1. Questionnaire

No. Responden (diisi oleh penyelidik) ☐☐☐☐

BAHAGIAN C: PENGETAHUAN MENGENAI PENYAKIT KENCING TIKUS (LEPTOSPIROSIS)

10. Apakah penyakit bawaan tikus yang anda tahu?

____

11. Adakah anda pernah mengetahui tentang penyakit kencing tikus (leptospirosis)?

Ya ☐ Jika Ya, ke **soalan 12**

Tidak ☐ Jika Tidak, terus ke **BAHAGIAN D**

12. Dari sumber manakah anda mengetahui tentang penyakit kencing tikus?

Tandakan (/). **(Boleh pilih lebih dari satu)**

a.	Kawan / Jiran	
b.	Radio	
c.	Televisyen	
d.	Suratkhabar	
e.	Kakitangan Kesihatan (doktor, jururawat)	
f.	Majalah	
g.	Risalah oleh Kementerian Kesihatan	
h.	Internet	
i.	Pihak pengurusan di tempat kerja	
j.	Pelajar dan Kakitangan Universiti	
k.	Lain-lain (nyatakan):_______________	

No. Responden (diisi oleh penyelidik)

	PUNCA DAN MOD JANGKITAN	YA	TIDAK	TIDAK TAHU
13.	Leptosipirosis adalah penyakit yang disebabkan oleh kuman			
14.	Ia boleh disebarkan melalui haiwan ke manusia (zoonotik)			
15.	Leptospirosis boleh dijangkiti melalui luka			
16.	Leptospirosis boleh dijangkiti melalui makanan tercemar			
17.	Leptospirosis boleh dijangkiti melalui gigitan nyamuk			
18.	Individu boleh terkena jangkitan jika berjabat tangan dengan orang yang telah dijangkiti			
	**PETANDA KLINIKAL, GEJALA, KOMPLIKASI**	**YA**	**TIDAK**	
19.	Individu yang dijangkiti akan mengalami sakit-sakit otot (*myalgia*)			
20.	Individu yang dijangkiti akan mengalami demam kuning (*jaundice*)			
21.	Individu yang dijangkiti bebas dari sebarang gejala penyakit			
22.	Penyakit ini boleh menyebabkan kematian			
23.	Penyakit ini boleh menyebabkan kanser paru-paru			
24.	Penyakit ini boleh menyebabkan kegagalan buah pinggang			
25.	Penyakit ini boleh menyebabkan kerosakan hati			
26.	Penyakit ini boleh menyebabkan kencing manis			
	**RISIKO**	**YA**	**TIDAK**	
27.	Makan sambil bekerja berisiko mendapat leptospirosis			
28.	Minum sambil bekerja berisiko mendapat leptospirosis			
29.	Merokok sambil bekerja berisiko mendapat leptospirosis			
30.	Pekerja Majlis Perbandaran tidak termasuk dalam kumpulan berisiko			
	**RAWATAN DAN PENCEGAHAN**	**YA**	**TIDAK**	
31.	Penyakit ini boleh dirawat			
32.	Penyakit ini boleh dikesan melalui pemeriksaan darah			
33.	Mengekalkan kebersihan di kawasan rumah boleh mencegah penyakit ini			
34.	Mengelak berjalan dalam lopak air / kawasan kerap banjir boleh mencegah penyakit ini			
35.	Memakai sarung tangan ketika bekerja boleh mencegah penyakit ini			
36.	Mengurangkan aktiviti berisiko tinggi berkaitan leptospirosis adalah langkah pencegahan penyakit			
37.	Penghapusan tikus dan kawasan pembiakan tikus adalah langkah pencegahan jangkitan			
38.	Simpan makanan dengan teratur boleh mencegah jangkitan			
39.	Elakkan berjalan dengan kaki ayam boleh mencegah jangkitan			

S2. Gamification module description.

Stations	Game description
You will be puzzlin'	Participants were asked to arrange two sets of A4 sized puzzles of any image related to leptospirosis such as disease symptoms and transmission within 2 min. Marks were given based on the number of puzzle pieces completed by all team members within 2 min.
Find *Leptospira* in the Flour	Participants had to find *Leptospira* (worm shaped sweets) hidden in the flour using their mouths. This section introduced how the bacteria look under the microscope. Marks were given based on the number of sweets successfully retrieved within 2 min.
Fishing Leptospirosis with chopsticks	One blindfolded participant, with the help of instructions from other team members, was given 1 min to correctly pick 10 pictures related to the symptoms of leptospirosis out of a bowl containing various kinds of images. Marks were given based on the number of the correct pictures he/she successfully retrieved from the bowl.
Target and Go - Dart Game	Participants were asked to throw a dart to the board aiming for pictures showing the transmission of leptospirosis attached to the board. They were given 1 min to complete this task. Marks were given based on the pictures they targeted.
Guess My Doodle	Ten cards each with a word describing prevention methods of leptospirosis were given to the participants. One participant had to create a drawing based on the word on the card he/she picked. The other team members had to correctly guess within 2 min.
Wink^2 Contagious	A group comprising 9-10 participants sat in a circle. One of the students was infected with leptospirosis. The healthy students in the group had to guess who was infected while avoiding being winked at by the infected person to avoid getting infected. The player who correctly identified the infected player was the winner.
Let's Charades!	Two groups of 10 participants each were arranged in pairs. Each pair was given one keyword related to the subject, for example leptospirosis. One participant held a card with a word on their forehead and the other participant had to act it out so that the individual with the card on their forehead could guess the word on his/her forehead. The partners were not allowed to say the word and had to act out what the keyword represented.
I'm Insta famous!	The participants were given three keywords related to the subject, e.g., leptospirosis symptoms and reservoirs. They had to act out the keywords, take a picture and upload it on Instagram with a touch of creativity. They also had to add a hash tag (#) of the subject (e.g., leptospirosis), tag the host's username and include a description of the image.
Video Parody–Artist in the making	Participants were asked to record a video parody using a trendy song with their own creative lyrics that described what they learned about leptospirosis. Marks were given based on creativity.
RPG Game	An RPG was developed by the host that covered three aspects: symptoms, treatment and prevention. The participants were given 3 min to play all levels (3 levels), and the player with the highest marks was the winner.
